# Supplementary material for: Observability constrains expertise-dependent kinematic readout in action prediction
Source: Cogn Res Princ Implic. 2026 May 17;11:38. doi: 10.1186/s41235-026-00735-9 (PMC13350761; doi:10.1186/s41235-026-00735-9)
Supplement: Supplementary file 1 — Additional file1 (DOCX 479 KB) [file 41235_2026_735_MOESM1_ESM.docx]

# Supplementary Material

## Kinematic Data Processing

Kinematic features were extracted from full-body motion capture as described in the main manuscript. The 17 anatomical keypoints were tracked across the entire serve sequence by OpenPose pretrained model. From the raw trajectories, we derived three types of kinematic information: (1) positional coordinates (horizontal and vertical), (2) joint angles (computed via arctangent), and (3) velocities (first-order temporal derivatives). To capture the temporal dynamics of the serve action, the motion sequence was divided into four equal time bins (T1, T2, T3, T4). This procedure preserves temporal information while allowing the encoding model to integrate evidence from different phases of movement. The final kinematic feature vector for each trial consisted of 8 base features repeated across 4 time bins, yielding 32 dimensions per trial: left/right shoulder x-coordinates, left/right elbow angles, and left/right wrist x/y-velocities.

## Hyperparameter Optimization

We used regularized logistic regression with elastic net penalty to map the kinematic feature vector to predictions about serve trajectory (Left or Right). The objective function minimized during model fitting was:

$$\mathcal{L}(\boldsymbol{\beta})=-\text{log}\mathcal{L}(\boldsymbol{\beta})+\lambda[(1-\alpha)\sum_{j=1}^{32} \beta_{j}^{2}+\alpha\sum_{j=1}^{32} |\beta_{j}|]$$

where $\lambda\geq0$ is the regularization parameter and $\alpha\in[0,1]$ is the mixing parameter. When $\alpha=0$, this reduces to ridge regression (L2 penalty); when $\alpha=1$, it becomes lasso regression (L1 penalty).

We performed extensive hyperparameter tuning using 5-fold stratified cross-validation with 50 repetitions across a grid of 100 lambda values (ranging from 10^-3 to 10^3). The procedure was repeated for three values of the elastic net mixing parameter alpha (0 = ridge, 0.5 = elastic net, and 1 = lasso).

Mean CV balanced accuracy and standard errors were computed by averaging across all participants and conditions (Figure S1). N non-zero coefficients reflect the average number of features retained across models. Based on comparable cross-validation performance across all three regularization schemes (difference in BalAcc < 0.001), we selected alpha = 0 (ridge regression) for all final models. This choice preserves weights for all 32 kinematic features, enabling complete comparison of weight vectors across observers without the confounding effects of feature selection. This approach is consistent with the previous study (Montobbio et al., 2022).


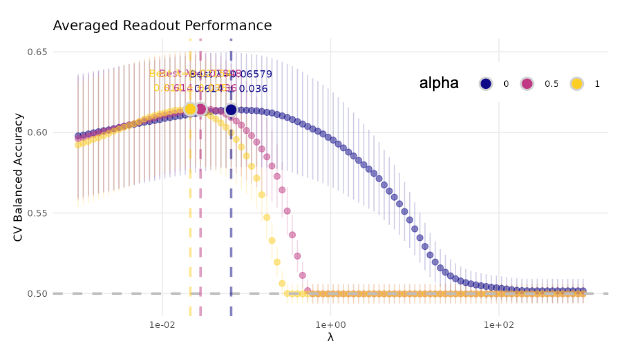


**Figure S1. CV Balanced Accuracy of readout models.** This figure shows the mean balanced accuracy (plus or minus 1 SE) from 5-fold cross-validation across 50 repetitions, plotted against the regularization parameter lambda on a logarithmic scale. Three curves are presented, corresponding to different values of the elastic net mixing parameter: (A) alpha = 0 (ridge regression), (B) alpha = 0.5 (elastic net), and (C) alpha = 1 (lasso regression).

## Encoding Model and Readout Model Weights

**
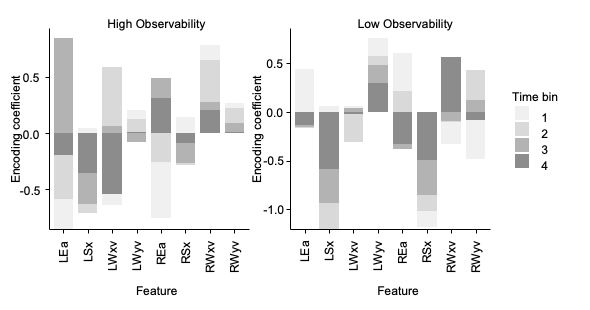
**

**Figure S2. Encoding coefficients**


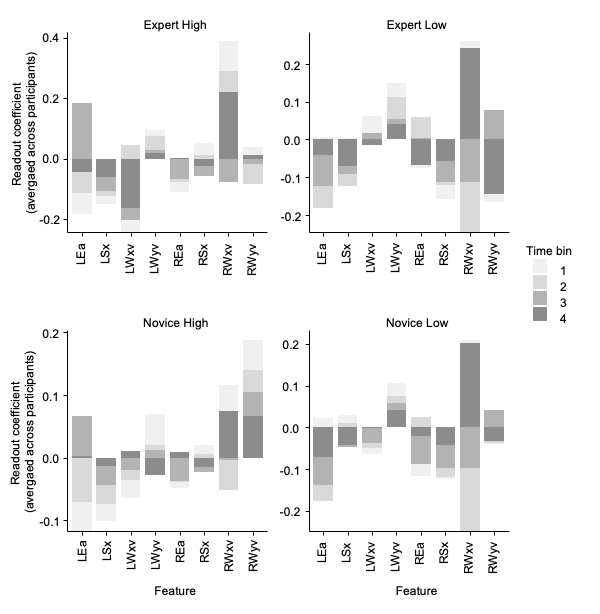


**Figure S3. Readout coefficients.**

# Supplementary Tables: Generalized Linear Mixed Model Results

## Table S1. Response Time as a Function of Model-Derived Confidence

Response time was modeled using a Gamma GLMM with log link function. Confidence was operationalized as |predicted probability − 0.5|.

**Model specification:** RT ~ Confidence × Expertise × Condition + (1 + Confidence + Condition | Participant)

### Table S1a. Type II Wald χ² Tests for Fixed Effects

| Effect | χ² | df | *p* |
| --- | --- | --- | --- |
| Confidence | 48.07 | 1 | < .001 |
| Condition | 0.62 | 1 | .431 |
| Expertise | 0.02 | 1 | .897 |
| Confidence × Condition | 1.79 | 1 | .181 |
| Confidence × Expertise | 0.40 | 1 | .525 |
| Condition × Expertise | 0.66 | 1 | .415 |
| Confidence × Condition × Expertise | 1.26 | 1 | .262 |

### Table S1b. Fixed Effects Estimates

| Parameter | *b* | SE | *z* | *p* |
| --- | --- | --- | --- | --- |
| (Intercept) | 7.420 | 0.023 | 316.17 | < .001 |
| Confidence | −0.147 | 0.021 | −6.91 | < .001 |
| Condition | 0.000 | 0.006 | 0.06 | .951 |
| Expertise | 0.007 | 0.023 | 0.31 | .756 |
| Confidence × Condition | 0.017 | 0.014 | 1.26 | .209 |
| Confidence × Expertise | −0.013 | 0.021 | −0.62 | .535 |
| Condition × Expertise | 0.001 | 0.006 | 0.19 | .850 |
| Confidence × Condition × Expertise | 0.015 | 0.014 | 1.12 | .262 |

*Note.* Estimates are on the log scale. Condition is sum-coded (High = 1, Low = −1). Expertise is sum-coded (Expert = 1, Novice = −1).

### Table S1c. Simple Slopes of Confidence on RT (Response Scale)

| Condition | Expertise | Slope (ms) | SE | *z* | *p* | 95% CI |
| --- | --- | --- | --- | --- | --- | --- |
| High | Expert | −221 | 59.4 | −3.72 | < .001 | [−338, −105] |
| High | Novice | −227 | 63.4 | −3.58 | < .001 | [−352, −103] |
| Low | Expert | −323 | 59.1 | −5.46 | < .001 | [−439, −207] |
| Low | Novice | −228 | 60.5 | −3.78 | < .001 | [−347, −110] |

### Table S1d. Pairwise Comparisons of Slopes

**By Expertise (within Condition):**

| Condition | Contrast | Δ Slope | SE | *z* | *p* |
| --- | --- | --- | --- | --- | --- |
| High | Expert − Novice | 6.1 | 86.9 | 0.07 | .944 |
| Low | Expert − Novice | −94.2 | 84.6 | −1.11 | .265 |

**By Condition (within Expertise):**

| Expertise | Contrast | Δ Slope | SE | *z* | *p* |
| --- | --- | --- | --- | --- | --- |
| Expert | High − Low | 101.5 | 61.5 | 1.65 | .099 |
| Novice | High − Low | 1.2 | 66.3 | 0.02 | .985 |

## Table S2. Readout Strength as a Function of Expertise and Condition

Readout strength (K·β) was modeled using a Gaussian GLMM with identity link.

**Model specification:** Readout Strength ~ Expertise × Condition + (1 + Expertise + Condition | Item) + (1 + Condition | Participant)

### Table S2a. Type II Wald χ² Tests for Fixed Effects

| Effect | χ² | df | *p* |
| --- | --- | --- | --- |
| Expertise | 9.42 | 1 | .002 |
| Condition | 3.18 | 1 | .074 |
| Expertise × Condition | 10.09 | 1 | .001 |

### Table S2b. Fixed Effects Estimates

| Parameter | *b* | SE | *z* | *p* |
| --- | --- | --- | --- | --- |
| (Intercept) | 0.271 | 0.051 | 5.36 | < .001 |
| Expertise | 0.114 | 0.027 | 4.25 | < .001 |
| Condition | 0.093 | 0.049 | 1.88 | .060 |
| Expertise × Condition | 0.078 | 0.025 | 3.18 | .001 |

*Note.* Condition is sum-coded (High = 1, Low = −1). Expertise is sum-coded (Expert = 1, Novice = −1).

### Table S2c. Estimated Marginal Means of Readout Strength

| Expertise | Condition | EMM | SE | *z* | *p* | 95% CI |
| --- | --- | --- | --- | --- | --- | --- |
| Expert | High | 0.558 | 0.077 | 7.25 | < .001 | [0.407, 0.709] |
| Expert | Low | 0.214 | 0.084 | 2.55 | .011 | [0.049, 0.378] |
| Novice | High | 0.172 | 0.064 | 2.70 | .007 | [0.047, 0.296] |
| Novice | Low | 0.144 | 0.091 | 1.58 | .115 | [−0.035, 0.323] |

###

### Table S2d. Simple Effects of Condition (within Expertise)

| Expertise | Contrast | Estimate | SE | *z* | *p* | 95% CI |
| --- | --- | --- | --- | --- | --- | --- |
| Expert | Low − High | −0.344 | 0.112 | −3.08 | .002 | [−0.564, −0.125] |
| Novice | Low − High | −0.028 | 0.109 | −0.25 | .800 | [−0.242, 0.186] |

###

### Table S2e. Simple Effects of Expertise (within Condition)

| Condition | Contrast | Estimate | SE | *z* | *p* | 95% CI |
| --- | --- | --- | --- | --- | --- | --- |
| High | Novice − Expert | −0.387 | 0.089 | −4.33 | < .001 | [−0.561, −0.212] |
| Low | Novice − Expert | −0.070 | 0.052 | −1.35 | .176 | [−0.171, 0.031] |

## Table S3. Encoding–Readout Coupling

Readout strength was regressed on encoding strength, with expertise and condition as moderators.

**Model specification:** Readout Strength ~ Encoding Strength × Expertise × Condition + (1 + Expertise | Item) + (1 + Condition | Participant)

### Table S3a. Type II Wald χ² Tests for Fixed Effects

| Effect | χ² | df | *p* |
| --- | --- | --- | --- |
| Encoding Strength | 8.17 | 1 | .004 |
| Expertise | 9.48 | 1 | .002 |
| Condition | 0.21 | 1 | .644 |
| Encoding Strength × Expertise | 12.87 | 1 | < .001 |
| Encoding Strength × Condition | 0.12 | 1 | .728 |
| Expertise × Condition | 6.01 | 1 | .014 |
| Encoding Strength × Expertise × Condition | 5.53 | 1 | .019 |

### Table S3b. Fixed Effects Estimates

| Parameter | *b* | SE | *z* | *p* |
| --- | --- | --- | --- | --- |
| (Intercept) | 0.017 | 0.096 | 0.18 | .861 |
| Encoding Strength | 0.115 | 0.035 | 3.30 | < .001 |
| Expertise | 0.014 | 0.035 | 0.40 | .691 |
| Condition | 0.031 | 0.096 | 0.32 | .745 |
| Encoding Strength × Expertise | 0.040 | 0.010 | 4.03 | < .001 |
| Encoding Strength × Condition | 0.001 | 0.035 | 0.03 | .978 |
| Expertise × Condition | 0.007 | 0.034 | 0.20 | .845 |
| Encoding × Expertise × Condition | 0.023 | 0.010 | 2.35 | .019 |

*Note.* Condition is sum-coded (High = 1, Low = −1). Expertise is sum-coded (Expert = 1, Novice = −1).

### Table S3c. Simple Slopes of Encoding Strength on Readout Strength

| Condition | Expertise | Slope | SE | *z* | *p* | *p*_Holm_ | 95% CI |
| --- | --- | --- | --- | --- | --- | --- | --- |
| High | Expert | 0.179 | 0.059 | 3.03 | .002 | .010 | [0.063, 0.296] |
| High | Novice | 0.053 | 0.055 | 0.97 | .332 | .332 | [−0.054, 0.160] |
| Low | Expert | 0.131 | 0.047 | 2.80 | .005 | .015 | [0.039, 0.223] |
| Low | Novice | 0.098 | 0.043 | 2.27 | .023 | .047 | [0.013, 0.182] |

### Table S3d. Pairwise Comparisons of Encoding–Readout Slopes

**By Expertise (within Condition):**

| Condition | Contrast | Δ Slope | SE | *z* | *p* | 95% CI |
| --- | --- | --- | --- | --- | --- | --- |
| High | Novice − Expert | −0.127 | 0.031 | −4.07 | < .001 | [−0.188, −0.066] |
| Low | Novice − Expert | −0.033 | 0.025 | −1.36 | .175 | [−0.082, 0.015] |

**By Condition (within Expertise):**

| Expertise | Contrast | Δ Slope | SE | *z* | *p* | 95% CI |
| --- | --- | --- | --- | --- | --- | --- |
| Expert | Low − High | −0.049 | 0.076 | −0.64 | .520 | [−0.197, 0.099] |
| Novice | Low − High | 0.045 | 0.070 | 0.64 | .520 | [−0.092, 0.181] |

## Notes on Model Estimation

1. **Software:** All GLMMs were fitted using the glmmTMB package (version 1.1.13) in R (version 4.5.1).
2. **Contrast coding:** Sum coding was applied to both Expertise (Expert = 1, Novice = −1) and Condition (High = 1, Low = −1). This coding ensures that main effects represent the average effect across levels of the other factor.
3. **Post-hoc corrections:** Holm-Bonferroni correction was applied to simple slope p-values for the encoding–readout coupling analysis (Table S3c). Other pairwise comparisons are uncorrected given the confirmatory nature of the hypotheses.
4. **Effect size interpretation:** For the RT model (Table S1), estimates on the log scale can be interpreted as proportional changes in RT; e.g., *b* = −0.147 for confidence corresponds to approximately 14% decrease in RT per unit increase in confidence.

## S4: Actor Confound and Within-Actor Observability

Because our design sampled high- and low-observability trials from different actors, we conducted three analyses to confirm that the observed effects reflect observability rather than actor-specific kinematic style.

### S4.1 Analysis 1: Actor as a factor in a three-way ANOVA

Per-actor encoding models were trained using ridge logistic regression (α = 0, λ selected via 5-fold CV) on each actor’s 60 items. Truth-corrected encoding strength was computed, and items were split at the within-actor median into High/Low encoding groups (30 items each per actor). d’ was analyzed using a 2 (Group) × 2 (Within-Actor Encoding Level) × 2 (Actor) mixed ANOVA.

**Table S4.1.** **Three-factor ANOVA on d’:**

| Effect | F(1,52) | *p* | η2 p |
| --- | --- | --- | --- |
| Group | 24.92 | < .001 | .324 |
| EncLevel | 0.00 | .988 | .000 |
| Actor | 8.03 | .007 | .134 |
| Group × EncLevel | 5.13 | .028 | .090 |
| Group × Actor | 6.91 | .011 | .117 |
| EncLevel × Actor | 0.06 | .801 | .001 |
| 3-way | 0.08 | .779 | .002 |


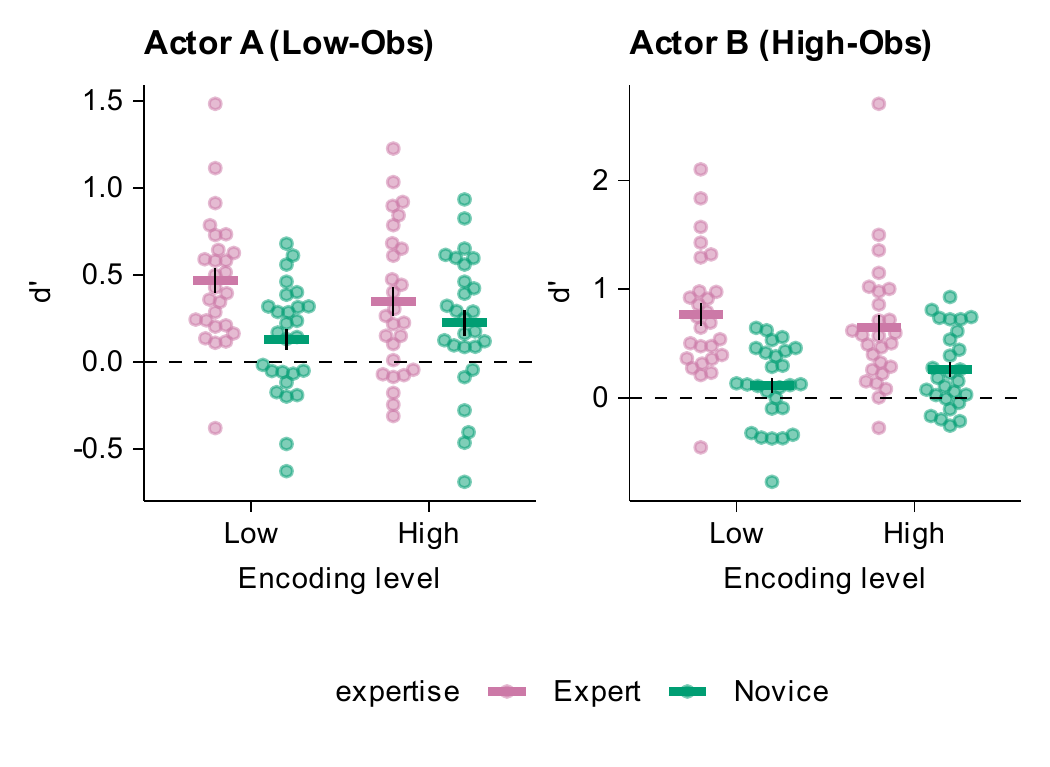


**Figure S4a. Within-actor d’ by encoding level and group.**

### S4.2 Analysis 2: Within-actor encoding–readout coupling

A trial-level GLMM was fit: readout_truth ~ enc_truth × expertise × actor + RE, with random effects selected by buildmer (BIC, backward elimination). All factors used sum-to-zero contrasts.

**Table S4.2. GLMM results:**

| Effect | *χ²* | *p* |
| --- | --- | --- |
| Encoding truth | 6.66 | .010 |
| Expertise | 4.86 | .028 |
| Enc truth × Expertise | 8.94 | .003 |
| Actor | 0.23 | .631 |
| Enc truth × Actor | 0.04 | .847 |
| Expertise × Actor | 4.50 | .034 |
| 3-way | 3.05 | .081 |


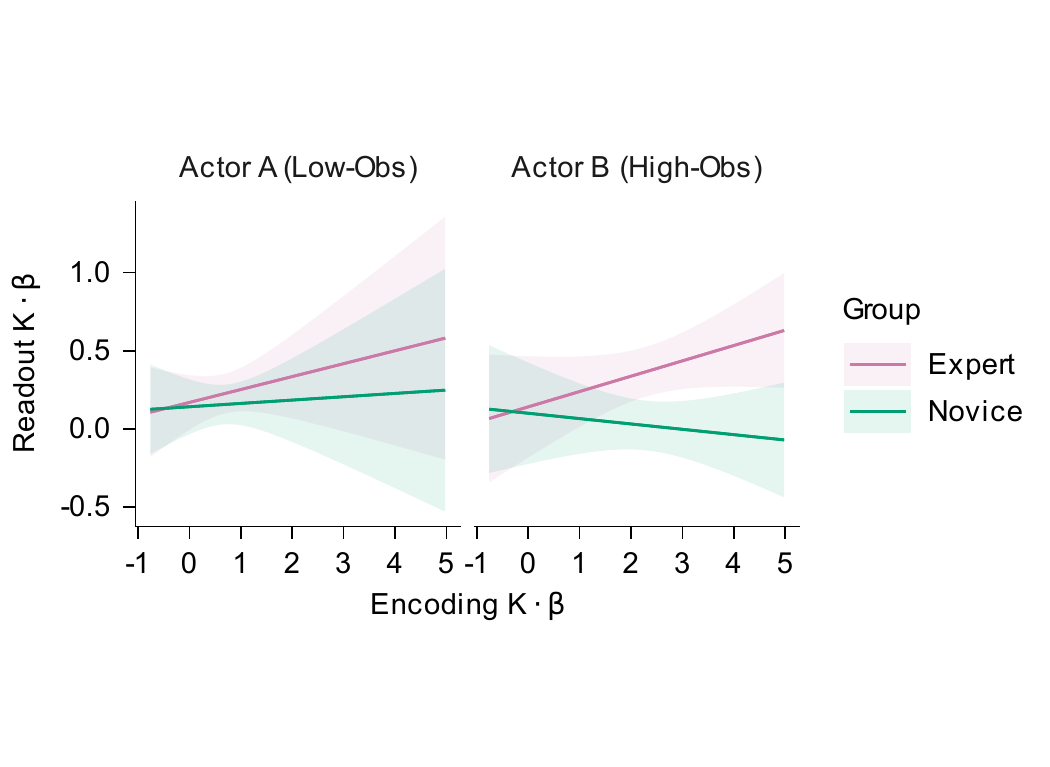


**Figure S4b. Within-actor encoding-readout coupling.**

### S4.3 Analysis 3: Combined encoding model

A single ridge logistic regression was trained on all 120 items (both actors pooled). Items were split at the global median into High/Low encoding groups, so encoding level is no longer confounded with actor identity. Item distribution: High = 25 Actor A + 35 Actor B; Low = 35 Actor A + 25 Actor B.

**Table S4.3.** **Two-factor ANOVA results on d’:**

| Effect | F(1,52) | *p* | η2 p |
| --- | --- | --- | --- |
| Group | 24.70 | < .001 | .322 |
| EncLevel | 34.74 | < .001 | .401 |
| Group × EncLevel | 2.52 | .119 | .046 |

| Group | Contrast | Estimate | *t*(52) | *p* | *d* |
| --- | --- | --- | --- | --- | --- |
| Expert | High − Low | +0.327 | 5.29 | < .001 | 1.03 |
| Novice | High − Low | +0.189 | 3.05 | .004 | 0.59 |

**Table S4.4 Encoding–readout coupling GLMM** (readout ~ encoding × expertise):

| Effect | *χ²* | *p* |
| --- | --- | --- |
| Encoding | 16.10 | < .001 |
| Expertise | 29.03 | < .001 |
| Encoding × Expertise | 85.59 | < .001 |

Per-participant slopes: Expert *M* = 0.199 ± 0.106; Novice *M* = 0.072 ± 0.086; *t* = 4.85, *p* < .001.

**Figure S4c. Combined model results: d’ and readout-encoding relationship.**

## S5: Encoding Model Stability

### S5.1 Cross-actor encoding validation

Training the encoding model on one actor and testing on the other yielded chance-level accuracy (50%), confirming actor-specific kinematic structure. A combined model trained on all 120 items achieved 87.5% balanced accuracy.

### S5.2 Encoding strength and bootstrap stability

Mean encoding K·β was computed for each observability condition.

**Table S5 Bootstrapped results of encoding strength**

| Observability | M | SD | 95% CI | *t* | *p* |
| --- | --- | --- | --- | --- | --- |
| High (Actor B) | 2.601 | 1.090 | [2.320, 2.883] | 18.49 | < .001 |
| Low (Actor A) | 0.711 | 0.726 | [0.523, 0.898] | 7.59 | < .001 |

High > Low: *t*(102.7) = 11.18, *p* < .001. 1,000 bootstrap resamples per actor confirmed stability: item-level encoding strengths correlated well between original and bootstrap models (Actor A: *r* = .785; Actor B: *r* = .819), and 78–82% of items maintained their High/Low classification.


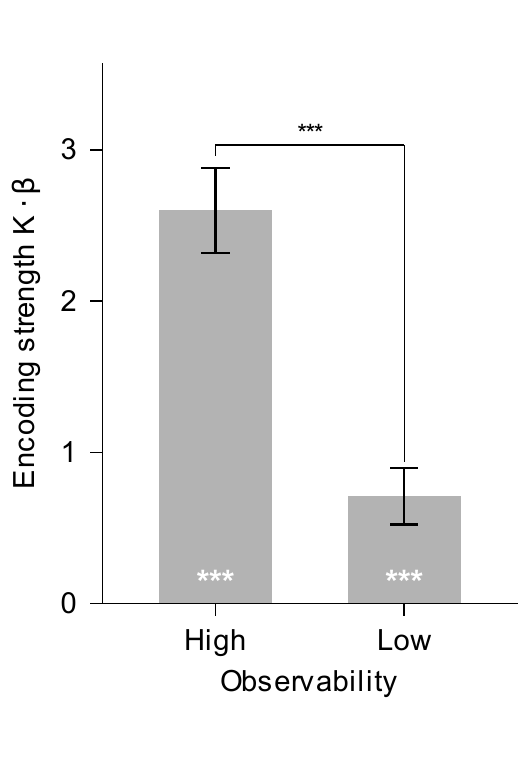


**Figure S5. Encoding strength per observability condition**
